# Glycolysis of Poly(Ethylene Terephthalate) Using Biomass-Waste Derived Recyclable Heterogeneous Catalyst

**DOI:** 10.3390/polym13010037

**Published:** 2020-12-24

**Authors:** Samson Lalhmangaihzuala, Zathang Laldinpuii, Chhakchhuak Lalmuanpuia, Khiangte Vanlaldinpuia

**Affiliations:** 1 Department of Chemistry, Pachhunga University College Campus, Mizoram University, Aizawl 796001, Mizoram, India; samsonzuala@gmail.com (S.L.); zathangdinpuii@gmail.com (Z.L.); empia34@gmail.com (C.L.); 2 Department of Chemistry, Mizoram University, Aizawl 796004, Mizoram, India

**Keywords:** biomass-waste, depolymerisation, glycolysis, heterogeneous catalyst, orange peel ash, PET waste

## Abstract

Plastic production has increased by almost 200-fold annually from 2 million metric tons per year in 1950s to 359 million metric tons in 2018. With this rapidly increasing production, plastic pollution has become one of the most demanding environmental issues and tremendous efforts have been initiated by the research community for its disposal. In this present study, we reported for the first time, a biomass-waste-derived heterogeneous catalyst prepared from waste orange peel for the depolymerisation of poly(ethylene terephthalate) (PET) to its monomer, bis(2-hydroxyethyl terephthalate) (BHET). The prepared orange peel ash (OPA) catalyst was well-characterised using techniques such as IR, inductively coupled plasma (ICP)-OES (Optical Emission Spectrometry), XRD, X-ray fluorescence (XRF), SEM, energy-dispersive X-ray spectroscopy (EDX), TEM, BET (Brunauer-Emmett-Teller) and TGA. The catalyst was found to be composed of basic sites, high surface area, and a notable type-IV N_2_ adsorption–desorption isotherm indicating the mesoporous nature of the catalyst, which might have eventually enhanced the rate of the reaction as well as the yield of the product. The catalyst completely depolymerises PET within 90 min, producing 79% of recrystallised BHET. The ability of reusing the catalysts for 5 consecutive runs without significant depreciation in the catalytic activity and its eco- and environmental-friendliness endorses this protocol as a greener route for PET recycling.

## 1. Introduction

Polyethylene terephthalate (PET) is a thermoplastic polyester formed by the condensation of terephthalic acid and ethylene glycol [1]. Due to its resistance to water, high strength to weight ratio, safety and widespread availability, it is extensively used in the production of textile fibres, bottles and films [2]. However, with increased use in the bottling and packaging industry, this non-biodegradable plastic waste skyrocketed and has developed into a serious global environmental issue [3,4]. It was reported that plastic packaging including PET accounts for 36% of global plastic productions (77.5 million metric tons), out of which 46% (35.7 million metric tons) is either incinerated or cast-off into the environment [3,4,5,6]. Considering the negative impact of plastic waste on living organisms and the ecosystem, it has become one of the major pollutants and the disposal and recycling of post-consumer plastic gained huge attention to the scientific community [7,8]. Moreover, the durability of plastics makes the recycling of PET products in great demand as it cannot be broken down easily by microorganisms in nature [9].

PET recycling can be carried out using mechanical or chemical processes [10]. Mechanical recycling follows sorting, washing and drying, size reduction, melt filtration and reforming of the plastics. In contrast, chemical recycling involves depolymerisation, purification and then re-polymerisation. Of the two processes, mechanical recycling is more practised but results in the degradation of the plastic properties while high-quality end-products can be achieved using the chemical recycling method, allowing them to re-polymerize to a virgin-grade plastic [11]. Depolymerization of PET through the chemical process can be achieved through solvolytic chain cleavage, which produces its monomers or oligomers. Moreover, this chemical recycling includes various methods such as glycolysis, methanolysis, hydrolysis, ammonolysis, and aminolysis, which are usually carried out at a high temperature and in the presence of catalysts [12]. 

Glycolysis of PET using ethylene glycol involves breaking up of the ester linkage and then the formation of bis(2-hydroxyethyl terephthalate) (BHET) monomer through transesterification. Factors such as temperature, time, solvent and catalyst loading and particle sizes have been reported to influence the outcome of the reactions [13]. Kinetic study of the glycolytic process revealed that complete depolymerization is impossible to achieve without the presence of a catalyst and required a longer reaction time. So, different types of catalysts including metal-based mixed oxides spinel [14], metal salts such as chlorides [15], acetates [16,17,18,19], carbonates [20] and sulphates [21,22], nitrogen-based organic acids [23], metal oxides [24], titanium phosphate [25], zeolites [26,27], ionic liquids [28,29,30], nanoparticles [31,32,33] and organocatalysts [34,35] were explored and reported to catalyse this transformation. Some of these methods endorse high yield of the monomer and short reaction time, but still present several shortcomings such as high cost of the catalysts, possible metal contamination in the final product, low yield or poor selectivity of the monomer and tedious purification process. 

Global production of agro-waste biomass was reported to be about 998 million tons with an increased rate of 5–10% per annum [36]. Many of these wastes ended up in landfills that pollute superficial and ground waters, and when amalgamated into the soil, they can invigorate the production and release of greenhouse gases such as NO and NO_2_ into the atmosphere [37]. Though large quantities of this biomass generated are utilized for the production of goods such as biogas, biochemicals, biofuels, bio-sorbent, animal feeds and biofertilizers, the transformation of these residues to value-added products could greatly improve solid waste management and reduce waste from the environment [38,39,40]. Recently, the use of these waste biomass-derived heterogeneous catalysts for promoting different types of reactions have gained propulsion as they are easily available, environmental-friendly, efficient and cost-effective [36,41]. They are mainly used for the production of biodiesel [36,42,43] and their efficacy as a catalyst in Henry reaction [44,45], Sonogashira reaction [46], Suzuki-Miyaura coupling reaction [47], Knoevenagel reaction [48], Dakin reaction [49], peptide bond formation [50] and *aza*-Michael reaction [51] was also explored. 

Meanwhile, the production of orange (*Citrus sinensis*) represents more than half of the total citrus fruit production worldwide with an estimate of 73.31 million tons of oranges produced in 2017 [52]. Though orange peel (OP) contains some important by-products and was traditionally used to obtain essential oils and flavouring compounds [39], such a level of consumption generates a huge amount of orange peel waste, which eventually ends up in landfills [53]. More recently, efforts have been made to utilize OP waste either as a carbon activated for catalyst support [54], as a heterogeneous catalyst for chemical transformations [55,56] and for the production of low-cost biodiesel [57,58]. However, to our knowledge, there is no report of biomass waste-derived heterogeneous catalyst for the depolymerization of PET waste. As to follow-up our interest in the field, we wish to report orange peel ash (OPA) as an efficient, simple and reusable heterogeneous catalyst for glycolysis of post-consumed PET bottles.

## 2. Experimental

### 2.1. Materials and Methods

PET beverage bottles were procured from the local market (Aizawl, Mizoram, India). It was washed with distilled water, dried and then shredded into 1 mm squares. Ethylene glycol (EG), commercially available BHET and methanol were purchased from Sigma-Aldrich (Steinheim, Germany) and used without further purification. Water was distilled and deionized prior to use.

FT-IR spectra were recorded on Spectrum BX FT-IR (Perkin-Elmer, Waltham, MA, United States) using KBr disks (υ_max_ in cm^−1^). Inductively coupled plasma (ICP)-OES (Optical Emission Spectrometry) was performed on iCAP 7600 ICP-OES Duo (Thermo Scientific, Waltham, MA, United States) with a wavelength range of 166–847 nm; it is fitted with high performance solid-state CID86 chip detector. Wavelength dispersive X-ray fluorescence (XRF) of OPA catalyst was investigated using PANalytical, Axios mAX. Scanning Electron Microscope (SEM) images and elemental compositions of the catalyst were recorded on JSM-6360 (Jeol, Akishima, Tokyo, Japan) and ESEM EDAX XL-30 (Phillips, Eindhoven, The Netherlands). Transmission Electron Microscope (TEM) image was recorded on JEM-2100, 200 kV instrument (Jeol, Akishima, Tokyo, Japan). Powder X-ray diffraction (XRD) was performed on Xpert MPD (Philips, Eindhoven, The Netherlands) to examine the crystalline composition of the catalyst. Analysis of thermal stability of the catalysts was obtained by TGA 4000 (Perkin Elmer, Waltham, MA, United States) under nitrogen atmosphere. Samples of 10 mg were heated from 30–995 °C at a heating rate of 10.00 °C/min. The N_2_ adsorption–desorption isotherm was recorded using Quanta chrome Nova-1000 surface area and porosity analyser. The samples were pre-treated at a degassing temperature of 150 °C for 10 hrs. HPLC (High-Performance Liquid Chromatography) analysis was performed on Waters 1525 binary pump using Spherisorb ODS2 5μm (Waters, Milford, MA, United States) 4.6 × 250 mm^2^ analytical column and Waters UV detector 2489, measuring at 254 nm. A mixture of methanol/water at a volume fraction of 70/30 was used as a mobile phase at a flow rate of 1 mL/min. ^1^H NMR and ^13^C NMR spectra were recorded on 400 MHz Avance III spectrometer (Bruker, Billerica, MA, United States) in deuterated dimethyl sulphoxide (DMSO-d_6_).

### 2.2. Preparation of the Catalyst

Oranges were purchased from local vendors in Aizawl, Mizoram, India. The peels were collected, washed thoroughly with distilled water and then dried in the open air for 3 days or in an oven at 80 °C for 12 h. Then, the dried orange peels were burnt in the open air. The greyish-white orange peel ash was collected and sifted using 100 BS and stored in a sample vial at room temperature. To study the composition, morphology and structure of the prepared catalysts, FT-IR, ICP, XRD, XRF, SEM, energy-dispersive X-ray spectroscopy (EDX), TEM, BET (Brunauer-Emmett-Teller) and TGA analyses were performed [57].

### 2.3. Glycolysis of PET Waste

For each reaction, 480 mg of PET flakes were depolymerised using a certain amount of EG and OPA. The reaction mixture was placed in a 100 mL two-necked round bottom flask fitted with a thermometer and a condenser. The reaction mixture was placed in an oil bath at a temperature of 190 °C under atmospheric pressure, and the reaction was allowed to run until complete disappearance of PET flakes. After completion of the reaction, the catalyst was separated by filtration and washed with hot water (100 mL). The solution was cooled down and the mixture was stirred vigorously and then filtered to separate BHET and EG from water-insoluble oligomers and additives. The filtrate was reduced to a volume of about 40 mL and stored in a refrigerator at 2 °C, where a crystallised BHET product separated that was removed by filtration and dried in a hot air oven. The depolymerised product was characterised using IR, NMR and HPLC analyses to confirm the production of the monomer. The yield percentage of the main product was calculated using the following equation:

(1) $$\mathrm{Yield}\;\mathrm{of}\;\mathrm{BHET}\;\mathrm{monomer}\;\left(\%\right)\;=\;\frac{\mathrm{Actual}\;\mathrm{yield}\;\mathrm{of}\;\mathrm{BHET}\;\mathrm{monomer}}{\mathrm{Theoretical}\;\mathrm{Yield}\;\mathrm{of}\;\mathrm{BHET}}\;\times \;100\;\%$$

## 3. Results and Discussion

### 3.1. Characterisation of the Catalyst

Elemental content of the OPA was characterised by inductively coupled plasma (ICP) analysis and it gave the following result: K (32.598 ppm), Ti (0.256 ppm), Sn (0.220 ppm), Al (2.510 ppm), Fe (7.589 ppm), Mn (3.653 ppm), Cu (0.297 ppm), Zn (0.251 ppm), Cr (0.265 ppm), Ca (161.222 ppm), Na (15.215 ppm) and Ni (0.015 ppm). To further examine the composition of OPA, XRF analysis was performed (Table 1) and it was observed that the catalyst was found to contain CaO (31.322 %), K_2_O (29.38 %), SO_3_ (15.792 %), MgO (7.037%) and P_2_O_5_ (5.188%) as major constituents. Other metal oxides such as Fe_2_O_3_, MnO and Al_2_O_3_ were also present in trace amounts. The results are well-coordinated with the reported analytical observations made by Changmai et al. [57].

**Figure 1:**
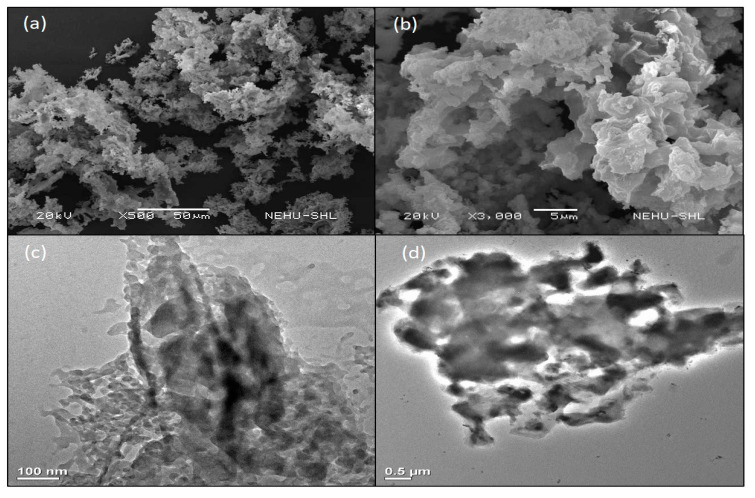
SEM [(**a**,**b**)] and TEM [(**c**,**d**)] images of OPA.

The structure and surface morphology of the catalyst were investigated by SEM and TEM analyses. Figure 1a,b shows SEM images with different magnifications, which revealed an agglomerated structure with high porosity. TEM images (Figure 1c,d) also exposed the squashy characteristic of the catalyst with mesoporous and microporous nature. The high absorbent nature of OPA which could provide a suitable morphology for adsorption of organic compounds and solvents along with the high basicity of the catalyst is believed to be responsible for its catalytic activity in PET glycolysis [56].

**Figure 2:**
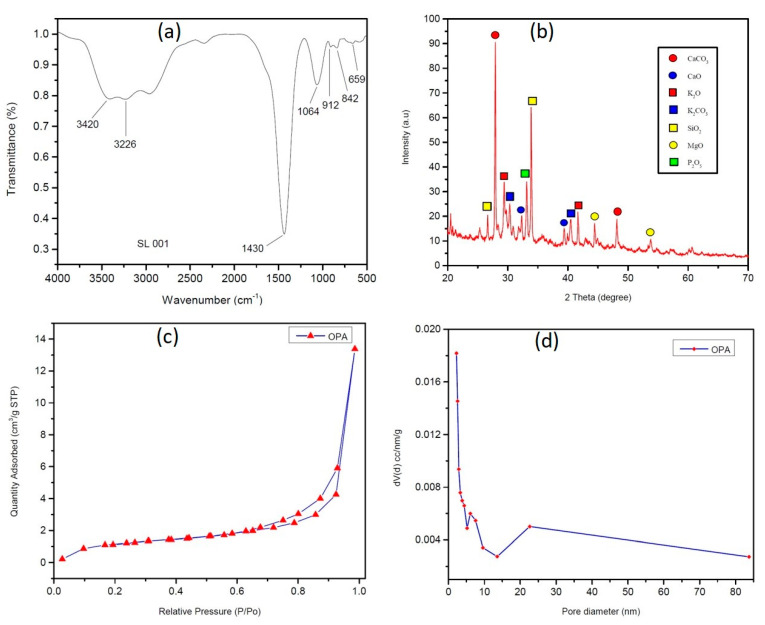
(**a**) FT-IR spectra of OPA (**b**) XRD data of OPA, (**c**) N_2_ adsorption–desorption isotherm, and (**d**) Barrett-Joyner-Halenda (BJH) pore size distribution analysis.

Fourier transform infrared spectroscopy was employed to investigate the functionalisation of OPA catalyst (Figure 2a). It exhibits a characteristic stretching vibration of O-H bond at 3420 cm^−1^ and 3226 cm^−1^, which may be due to the water molecule absorbed in the catalyst. The absorption peaks at approximately 1430 cm^−1^, 1064 cm^−1^ and 842 cm^−1^ were assigned to metal carbonate C–O stretching and bending frequency.

To investigate the crystalline nature of the OPA catalyst, powder X-ray diffraction studies were conducted. XRD of the OPA in Figure 2b indicated the presence of metal oxides and carbonates such as K_2_O, CaO, MgO, SiO_2_, K_2_CO_3_, CaCO_3_, etc. The presence of CaCO_3_ and CaO was confirmed by the presence of distinctive peaks at 2θ = 27.233, 48.177, 32.322, and 39.508 (JCPDS reference file No: 76–0606, 86–2341, 82–1691, and 28–0775). The presence of K_2_O and K_2_CO_3_ was also established by the presence of peaks at 2θ = 29.311, 41.605, 40.7082, and 30.222 (JCPDS reference no: 77–2176 and 87–0730). Furthermore, additional peaks at 2θ = 26.506 and 34.222 (SiO_2_, JCPDS reference no: 89–8936 and 89-3609), 2θ = 44.422 and 53.560 (MgO, JCPDS reference no: 30–0794 and 76–1363), and 2θ = 30.243 (P_2_O_5_, JCPDS reference no: 85–1120) were also observed. 

The N_2_ adsorption–desorption isotherm and pore size distribution analyses were carried out to investigate the surface area, pore volume and the average pore size of the catalyst (Figure 2c,d) and it was found to be 4.947 m^2^/g, 0.022 cm^3^/g and 25.22 Å, respectively. The N_2_ adsorption–desorption isotherm exhibits a typical type-IV isotherm, which is the characteristics of a mesoporous material [59].

**Figure 3:**
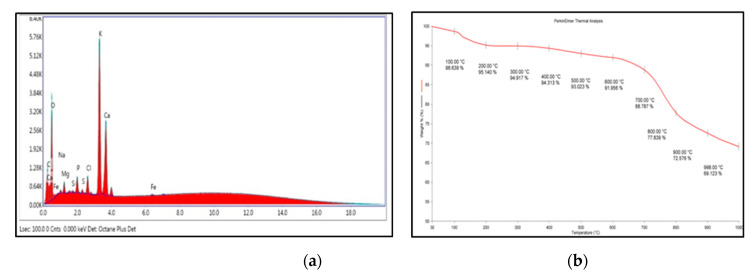
(**a**) Energy-dispersive X-ray spectroscopy (EDX) analysis of OPA, and (**b**) TGA thermogram of OPA.

Energy-dispersive X-ray spectroscopy (EDX) displayed the elemental composition of OPA catalyst (Figure 3a), indicating the presence of C, O, Mg, P, Cl, K and Ca. Table 2 shows the elemental distribution with weight % and atomic % in the catalyst.

The thermal stability of the OPA catalyst was examined using TGA analysis under N_2_ atmosphere in the range of 30–1000 °C. The first weight loss of 5% (Figure 3b) from about 30 °C to 200 °C was detected, which corresponds to the loss of water molecule present in the OPA catalyst. The second weight loss detected between 650 °C and 900 °C of about 21% might be due to the decomposition of the carbon moiety.

### 3.2. Optimisation of the Depolymerisation Condition

**Figure 4:**
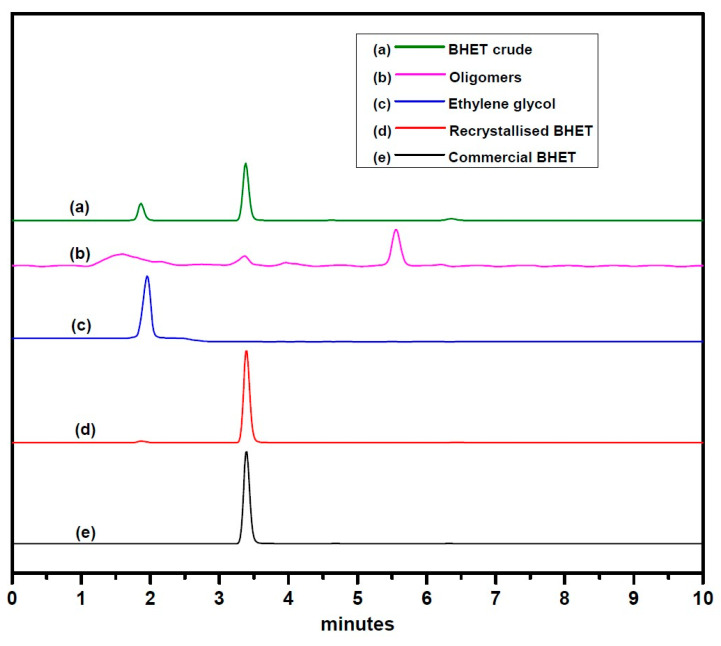
HPLC data of—(**a**) the crude product, (**b**) water-insoluble part, (**c**) ethylene glycol, (**d**) recrystallized bis(2-hydroxyethyl terephthalate) (BHET), and (**e)** commercially available BHET.

**Figure 5:**
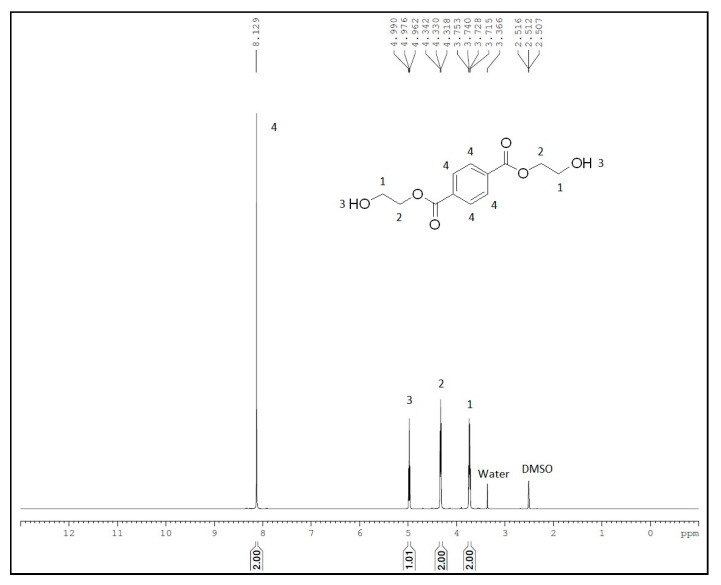
^1^H NMR spectra of recrystallised BHET.

Glycolysis of PET was carried out under atmospheric pressure in the presence of excess ethylene glycol at 190 °C. After the disappearance of PET flakes, the reaction mixture was filtered to separate the catalyst. Analysis of the final crude product by HPLC (Figure 4) using reversed-phase column revealed the presence of two smaller peaks at a retention time of 1.866 and 6.361 min, which might be due to the presence of EG and oligomers, respectively. A major peak at a retention time of 3.382 min is assigned to BHET monomer, which is consistent with the earlier report by Goh et al. [60]. HPLC analysis of the recrystallized product, commercially available BHET, EG and water-insoluble part revealed the formation of BHET as well as products other than the monomer (see Supplementary Material Figures S1–S5). Recrystallisation of the crude product with water affords pure BHET, which shows ^1^HNMR peak at δ = 2.51 ppm (DMSO), 3.74 ppm (C*H_2_*-OH), 4.33 ppm (COO-C*H_2_*), 5.00 ppm (-OH) and 8.09 ppm (Ar-H) and is in accord with the reported literature (Figure 5) [30,34]. Its ^13^C NMR data exhibit a characteristic peak of carbonyl carbon at δ = 165.12 ppm and aromatic carbons at δ =133.70 and 129.47 ppm. Resonances at δ = 66.99 (-O-CH_2_-CH_2_-OH) and 58.95 ppm (-O-CH_2_-CH_2_-OH) were also observed, which confirmed the formation of the desired product (see Supplementary Material Figure S7). The water-insoluble fraction was found to be composed mainly of BHET dimer, but small amount of other contaminants are likely to be present, as PET bottles generally contain small amount of these impurities for their improved moldability (see Supplementary Material Figures S8 and S9) [61,62].

In its FT-IR spectra, four prominent peaks were observed at υ = 3420 cm^−1^, 1726 cm^−1^, 1275 cm^−1^ and 1100 cm^−1^, which can be attributed to the presence of -OH group, carbonyl group and two C-O stretching vibrations, respectively (see Supplementary Material Figure S10). To enhance the reaction condition for the selective formation of BHET monomer, the effect of different parameters such as catalyst loading, EG content, reaction time and reaction temperature were investigated and the results are depicted in Table 3. 

#### 3.2.1. Effect of Catalyst Loading

**Scheme 1:**
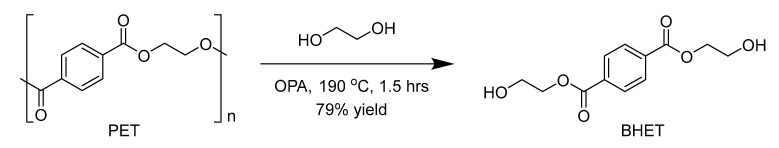
Depolymerisation of poly(ethylene terephthalate) (PET).

It was observed that varying the amount of catalyst concentration significantly influences the outcome of the BHET yield (Table 3, entry 1–5). Experiments were conducted to evaluate the effect of catalyst concentration for PET glycolysis using 14 equivalents of EG at 190 °C for 1 h under atmospheric pressure (Scheme 1). The catalyst displayed rapid degradation of PET, and the disappearance of PET flakes was used as an observable indicator for the end of the reaction [62]. Analysis of the crude products by HPLC analysis revealed the formation of BHET monomer as well as its dimer and/or other oligomers (see Supplementary Material Figures S1–S5). It was found that with as little as 4 wt % of the catalyst, glycolysis was completed within 1 h (Table 3, entry 1). However, purification of the crude product in water and separation of the recrystallized monomer revealed that increasing the amount of the catalyst loading to 10 wt% gave the most effective performance (Table 3, entry 3), and we chose this concentration to determine optimal condition for other parameters.

#### 3.2.2. Impact of EG Concentration

It was described earlier that an equilibrium between BHET and other oligomers, specifically its dimer, exists during glycolysis, and an excess amount of reagent loading is required to reduce the formation of species other than BHET monomer [62,63]. Our results are consistent with these reports and with an increase in the ratio of EG to PET, the yield of BHET monomer increases. The reaction proceeded smoothly even with 6 eq. of EG (Table 3, entry 6), but the final BHET yield was substantially lower when compared with the use of higher equivalents. There is also a clear correlation between an increased yield of the water-soluble product when using a lower PET/EG ratio, and the recrystallised products usually contain a noticeable amount of the dimer and/or oligomers of BHET. The best result was obtained with 16 eq. of EG, affording pure monomer with 78% yield within one hour (Table 3, entry 8). Increasing the reagent loading above this quantity did not provide any noticeable enhancement (Table 3, entry 9).

#### 3.2.3. Influence of Reaction Time and Temperature

**Figure 6:**
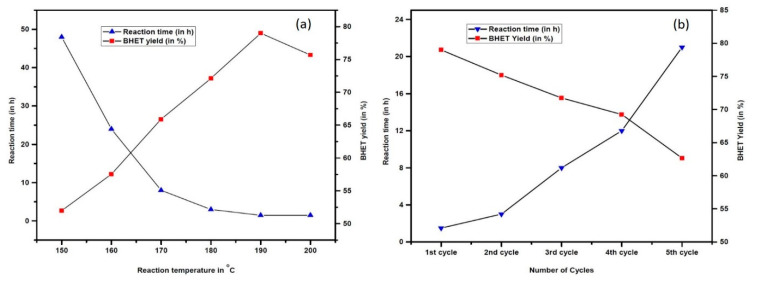
(**a**) Effect of reaction time on PET depolymerisation. The reaction was conducted using 2.5 mmol of PET, 16 equivalents of EG and 10 wt % of OPA, and (**b**) Reusability of OPA catalyst. The reaction was conducted using 2.5 mmol of PET, 16 equivalents of EG and 10 wt% of OPA catalyst.

It can be seen from Table 3, entry 8, 10 and 11 that the conversion of PET and the yield of BHET increase ascendingly by prolonging the reaction time. The highest yield of BHET was achieved after 90 min of degradation time, yielding 79% of BHET when the reaction temperature and EG loading were fixed at 190 °C and 16 eq., respectively (Table 3, entry 11). The reaction could be carried out even at a reduced temperature (150 °C) but required longer reaction time (Figure 6a). Increasing the reaction temperature beyond 190 °C results in the augmented formation of a water-insoluble product (see Supplementary Material Table S1).

#### 3.2.4. Recycling of EG and Catalyst 

Assessment of the viability of catalyst and reagent recycling has become the emphasis of most chemists for reasons of economic and environmental applicability. So, the reusability and recovery of the OPA catalyst and EG under our optimised condition was investigated for further glycolysis of PET (see Supplementary Material Table S2). After filtration, the catalyst was washed with methanol and then dried in an oven at 80 °C for 5 h. Usually, there was a loss of about 5–10% catalyst, which was recompensed with fresh catalyst. It was noticed that the catalyst selectivity towards BHET formation reduced marginally even after the repetitive cycle but the reaction time progressively decreases (Figure 6b). This increase in reaction time and decrease in the yield within each consecutive cycle might be due to the leaching of the catalyst’s active sites during the reaction and purification process. After separation of the BHET crystal, the aqueous phase was evaporated under vacuum at 60 °C to afford the unreacted EG and then stored in an oven at 50 °C for 12 h. This recovered residual reagent could be used for subsequent runs. 

After the 5th cycle, FT-IR spectra, SEM and TEM images along with EDX analysis of the reused catalysts were obtained to analyse variations in their functionality, morphology, structure and chemical contents. FT-IR spectra of the recycled OPA catalyst exhibited almost no significant changes from that of the fresh OPA catalyst (see Supplementary Material Figure S11). EDX analysis displays a substantial loss of potassium (K) wt % from 21.32% to 0.48% after the 5th cycle (see Supplementary Material Figure S12). SEM and TEM images were seen to exhibit an uneven morphological structure with mesoporous characteristic similar to that in the fresh catalyst (see Supplementary Material Figures S13 and S14).

## 4. Conclusions

In summary, we reported that PET polyester can be rapidly and completely depolymerised to its monomer BHET using eco-friendly, bio-derived solid heterogeneous orange peel ash catalyst, furnishing 79% yield of the recrystallised product. The glycolytic degree of conversion of post-consumer PET to BHET was greatly influenced by reaction parameters such as catalyst concentration, reaction time, reaction temperature and PET/EG ratio. Furthermore, the presence of medium and strongly basic sites such as oxides of potassium and calcium in our catalyst might have affected the reactivity of the transesterification reaction. The catalyst is non-toxic, renewable, readily accessible and cost-free and hence offers an alternative greener technique for chemical recycling of PET waste analogous to other approaches.

## Figures and Tables

**Table 1:** Determination of orange peel ash (OPA) composition using X-ray fluorescence (XRF) analysis.

Sl. No.	Compound	Concentration (%)
1	Na_2_O	0.108
2	MgO	7.037
3	Al_2_O_3_	0.123
4	SiO_2_	1.115
5	P_2_O_5_	5.188
6	SO_3_	15.792
7	K_2_O	30.866
8	CaO	32.868
9	Cr_2_O_3_	0.129
10	MnO	0.268
11	Fe_2_O_3_	2.152
12	CuO	0.201
13	ZnO	0.223
14	Rb_2_O	0.579
15	SrO	1.855
16	BaO	0.465

**Table 2:** EDX data for elemental composition of OPA.

Sl No.	Elements	Weight %	Atomic %
1	C	6.86	11.66
2	O	51.73	65.97
3	Na	0.67	0.60
4	Mg	1.30	1.09
5	Si	0.10	0.07
6	P	1.60	1.06
7	S	0.30	0.19
8	Cl	1.82	1.05
9	K	21.32	11.13
10	Ca	13.73	6.99
11	Fe	0.57	0.21

**Table 3:** Optimisation of BHET ^a^.

Sl. No.	Catalyst Loading (mg)	EG Loading (in g)	Time (h)	Conversion (%)	BHET Yield (%)
1	20	2	1	100	63
2	30	2	1	100	71
3	50	2	1	100	75
4	70	2	1	100	69
5	100	2	1	100	71
6	50	1	1	100	61
7	50	1.5	1	100	73
8	50	2.5	1	100	78
9	50	3	1	100	76
10	50	2.5	0.5	80	54
**11**	**50**	**2.5**	**1.5**	**100**	**79**
12	50	2.5	2	100	76
13	50	2.5	2.5	100	71

^a^ Reactions were conducted at 190 °C under atmospheric pressure.

## Supplementary Materials

The following are available online at https://www.mdpi.com/2073-4360/13/1/37/s1, Table S1: Effect of reaction temperature on the degradation of PET, Table S2: Reusability of OPA catalyst, Figure S1: HPLC data of the crude product, Figure S2: HPLC data of recrystallised BHET, Figure S3: HPLC data of commercially available BHET, Figure S4: HPLC data of ethylene glycol, Figure S5: HPLC data of water-insoluble part, Figure S6: ^1^H NMR data of recrystallised BHET, Figure S7: ^13^C NMR spectra of BHET, Figure S8: ^1^H NMR data of water-insoluble part, Figure S9: ^13^C NMR data of water-insoluble part, Figure S10: IR spectra of recrystallised BHET, Figure S11: IR spectra of recovered OPA catalyst after the 5th cycle, Figure S12: EDX data of recovered catalyst after the 5th cycle, Figure S13: SEM images of recovered OPA after the 5th cycle, Figure S14: TEM image of recovered OPA after the 5th cycle.
