# Supplementary material for: Glycolysis of Poly(Ethylene Terephthalate) Using Biomass-Waste Derived Recyclable Heterogeneous Catalyst
Source: Polymers (Basel). 2020 Dec 24;13(1):37. doi: 10.3390/polym13010037 (PMC7794874; doi:10.3390/polym13010037)

Glycolysis of poly(ethylene terephthalate) using biomass-waste derived recyclable heterogeneous catalyst

Samson Lalhmangaihzuala [1,2], Z.T Laldinpuii [1,2], C. Lalmuanpuia[1,2] and K. Vanlaldinpuia [1,*]

[1] Department of Chemistry, Mizoram University, Pachhunga University College Campus, Aizawl, Mizoram, India- 796001; samsonzuala@gmail.com (S. L. H); zathangdinpuii@gmail.com (Z. T. L. D); empia34@gmail.com (C. L. M. P).

[2] Department of Chemistry, Mizoram University, Aizawl, Mizoram, India – 796004.

[*] Correspondence: mapuiakhiangte@gmail.com; Tel.: +91 9862086476

SUPPLEMENTARY MATERIAL

General procedure for depolymerization of PET: A two necked 100 mL round bottom flask fitted with thermometer and reflux condenser was loaded with 480 mg (2.5 mmol) of PET flakes, 2.25 mL of ethylene glycol and 50 mg of OPA. The mixture was immersed in an oil bath and the reaction was carried out at 190 °C in an atmospheric pressure. After completion of the reaction (1.5 hrs), the catalyst was separated quickly *via* filtration and then washed with 100 mL of hot deionized water. After the mixture was cooled down, the mixture was stirred vigorously. The white precipitate insoluble in water was filtered. The filtrate was then concentrated to about 40 mL and stored in a refrigerator at 2 °C for 12 hrs. The white crystalline product formed in the filtrate was separated, dried and then weighed (79% yield).

Table S1: Effect of reaction temperature on the degradation of PET[a]

Sl. No	Reaction temperature (°C)	Reaction time (in h)	BHET yield in mg
1	150	48	51.98
2	160	24	57.55
3	170	8	65.89
4	180	3	72.12
**5**	**190**	**1.5**	**79.00**
6	200	1.5	75.68

[a]Reaction condition: 2.5 mmol of PET, 16 equivalents of EG, 10 wt % of OPA.

Table S2: Reusability of OPA catalyst[a]

No. of cycle	Time	BHET yield %
**1st cycle**	**1.5**	**79.00**
2nd cycle	3	75.20
3rd cycle	8	71.75
4th cycle	12	69.25
5th cycle	21	62.66

[a]Reaction condition: 2.5 mmol of PET, 16 equivalents of EG, 10 wt % of OPA, 190 °C.

Figure S1: HPLC data of the crude product

Pachhunga University College

Breeze 2 HPLC System

Project Name:	UV Project
Reported by User:	Breeze user (Breeze)

SAMPLE INFORMATION

Sample Name:	bhet crude1.5 hr 70 mg cat1	Acquired By:	Breeze
Sample Type:	Unknown	Date Acquired:	05-02-2020 15:32:36 IST
Vial:	1	Acq. Method:	bhet
Injection #:	1	Date Processed:	27-10-2020 16:27:47 IST
Injection Volume:	20.00 ul	Channel Name:	W2489 ChA
Run Time:	10.00 Minutes	Sample Set Name:	

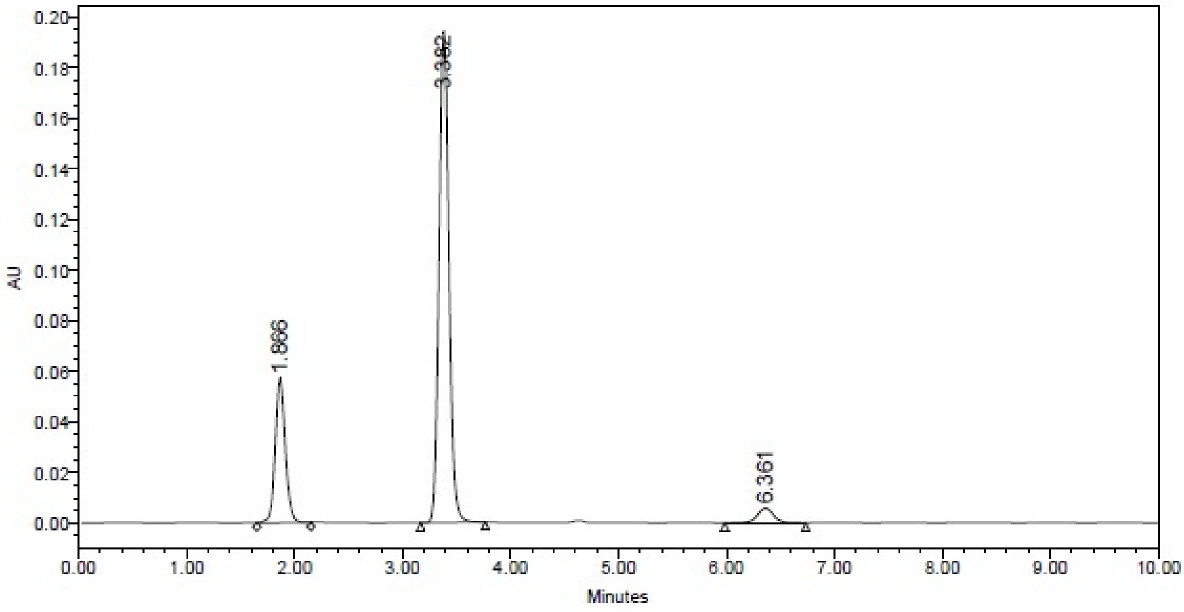

	RT (min)	Area (µV*sec)	% Area	Height (µV)	% Height
1	1.866	372664	22.24	57264	22.32
2	3.382	1240235	74.00	193445	75.40
3	6.361	63068	3.76	5841	2.28

Report Method: Untitled
Page: 1 of 1

Printed: 27-10-2020
16:28:15 Asia/Calcutta

Figure S2: HPLC data of recrystallized BHET

Pachhunga University College

Project Name: UV Project

Reported by User: Breeze user (Breeze)

SAMPLE INFORMATION

Sample Name:	bhet recrystallize1	Acquired By:	Breeze
Sample Type:	Unknow n	Date Acquired:	06-02-2020 14:18:55 IST
Vial:	1	Acq. Method:	bhet
Injection #:	1	Date Processed:	27-10-2020 16:47:28 IST
Injection Volume:	20.00 ul	Channel Name:	W2489 ChA
Run Time:	10.00 Minutes	Sample Set Name:	

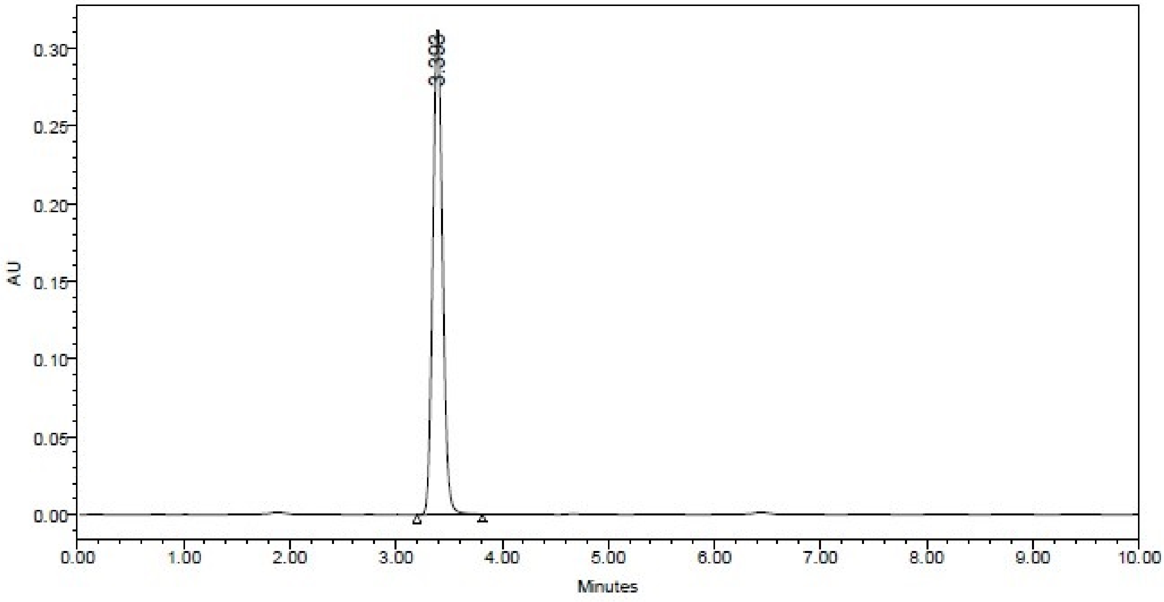

	RT (min)	Area (µV*sec)	% Area	Height (µV)	% Height
1	3.393	2015945	100.00	313590	100.00

Report Method: Untitled

Page: 1 of 1

Printed: 27-10-2020 16:47:51 Asia/Calcutta

Figure S3: HPLC data of commercially available BHET

Pachhunga University College

Project Name: UV Project

Reported by User: Breeze user (Breeze)

SAMPLE INFORMATION

Sample Name:	bhet 50	Acquired By:	Breeze
Sample Type:	Unknown	Date Acquired:	30-01-2020 15:22:22 IST
Vial:	1	Acq. Method:	bhet
Injection #:	1	Date Processed:	30-01-2020 16:05:36 IST
Injection Volume:	20.00 ul	Channel Name:	W2489 ChA
Run Time:	10.00 Minutes	Sample Set Name:	

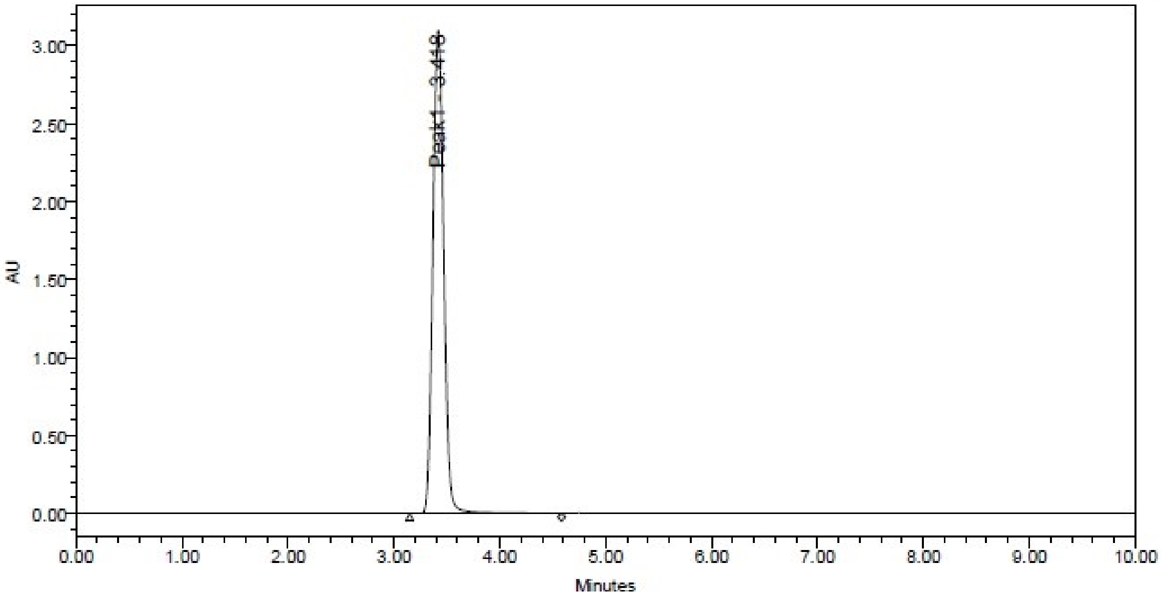

	Peak Name	RT (min)	Area (µV*sec)	% Area	Height (µV)	% Height
1	Peak1	3.418	22102893	100.00	3092909	100.00

Report Method: Untitled

Page: 1 of 1

Printed: 12-08-2020 14:58:26 Asia/Calcutta

Figure S4: HPLC data of ethylene glycol

Pachhunga University College

Project Name: UV Project

Reported by User: Breeze user (Breeze)

Breeze 2 HPLC System

SAMPLE INFORMATION

Sample Name:	ethylene glycol crude	Acquired By:	Breeze
Sample Type:	Unknow n	Date Acquired:	28-04-2020 12:31:11 IST
Vial:	1	Acq. Method:	bhet
Injection #:	1	Date Processed:	14-08-2020 17:37:10 IST
Injection Volume:	20.00 ul	Channel Name:	W2489 ChA
Run Time:	30.00 Minutes	Sample Set Name:	

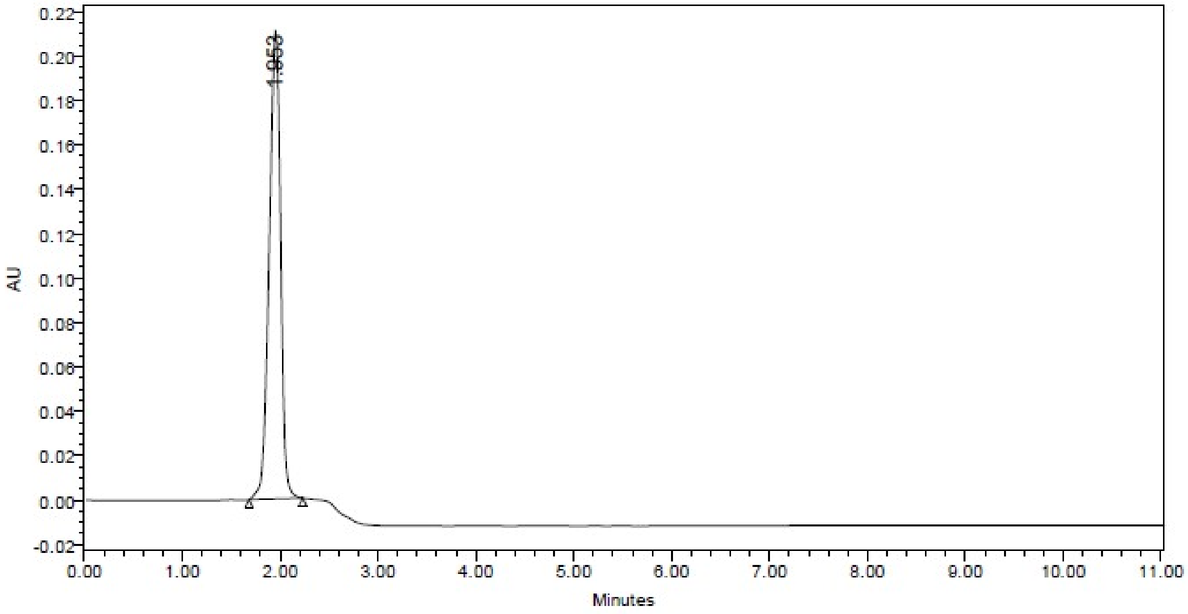

	RT (min)	Area (µV*sec)	% Area	Height (µV)	% Height
1	1.953	1680058	100.00	210724	100.00

Report Method: Untitled

Page: 1 of 1

Printed: 13-10-2020

17:05:04 Asia/Calcutta

Figure S5: HPLC data of water insoluble part

Pachhunga University College

Project Name: UV Project

Reported by User: Breeze user (Breeze)

Breeze 2 HPLC System

SAMPLE INFORMATION

Sample Name:	w ater insoluble pure1	Acquired By:	Breeze
Sample Type:	Unknow n	Date Acquired:	03-03-2020 18:56:08 IST
Vial:	1	Acq. Method:	bhet
Injection #:	1	Date Processed:	27-10-2020 11:56:15 IST
Injection Volume:	20.00 ul	Channel Name:	W2489 ChA
Run Time:	10.00 Minutes	Sample Set Name:	

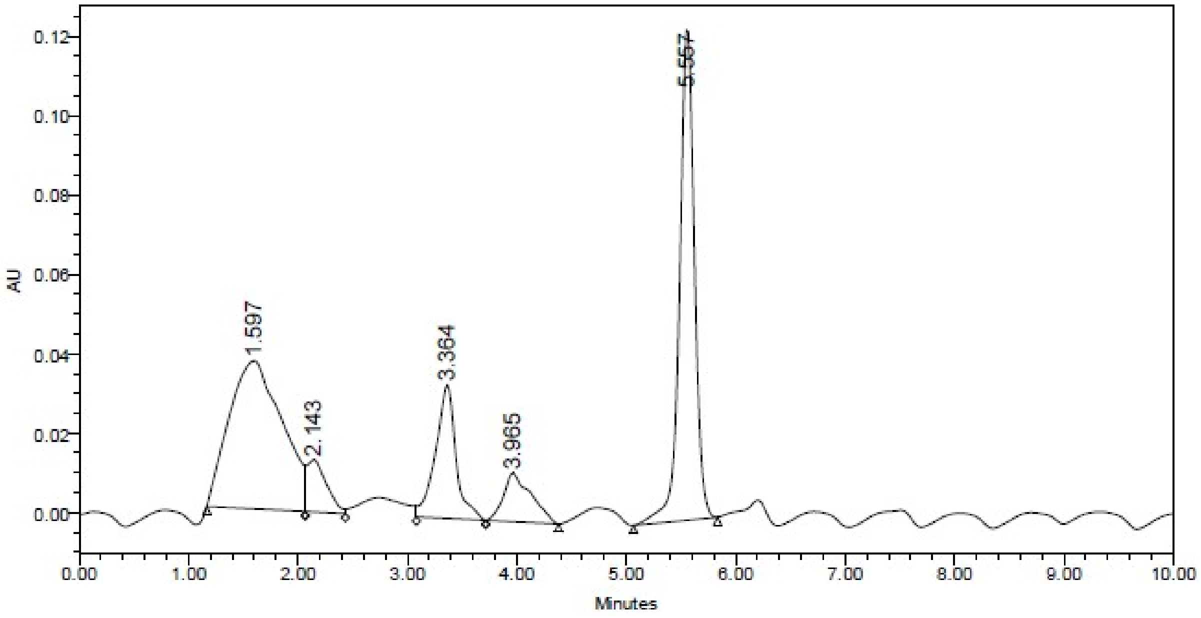

	RT (min)	Area (µV*sec)	% Area	Height (µV)	% Height
1	1.597	1253552	38.72	37366	16.93
2	2.143	165370	5.11	13177	5.97
3	3.364	434745	13.43	33707	15.27
4	3.965	215355	6.65	12220	5.54
5	5.557	1168261	36.09	124230	56.29

Report Method: Untitled
Page: 1 of 1

Printed: 27-10-2020
11:56:50 Asia/Calcutta

Figure S6: ^{1}H NMR data of recrystallized BHET

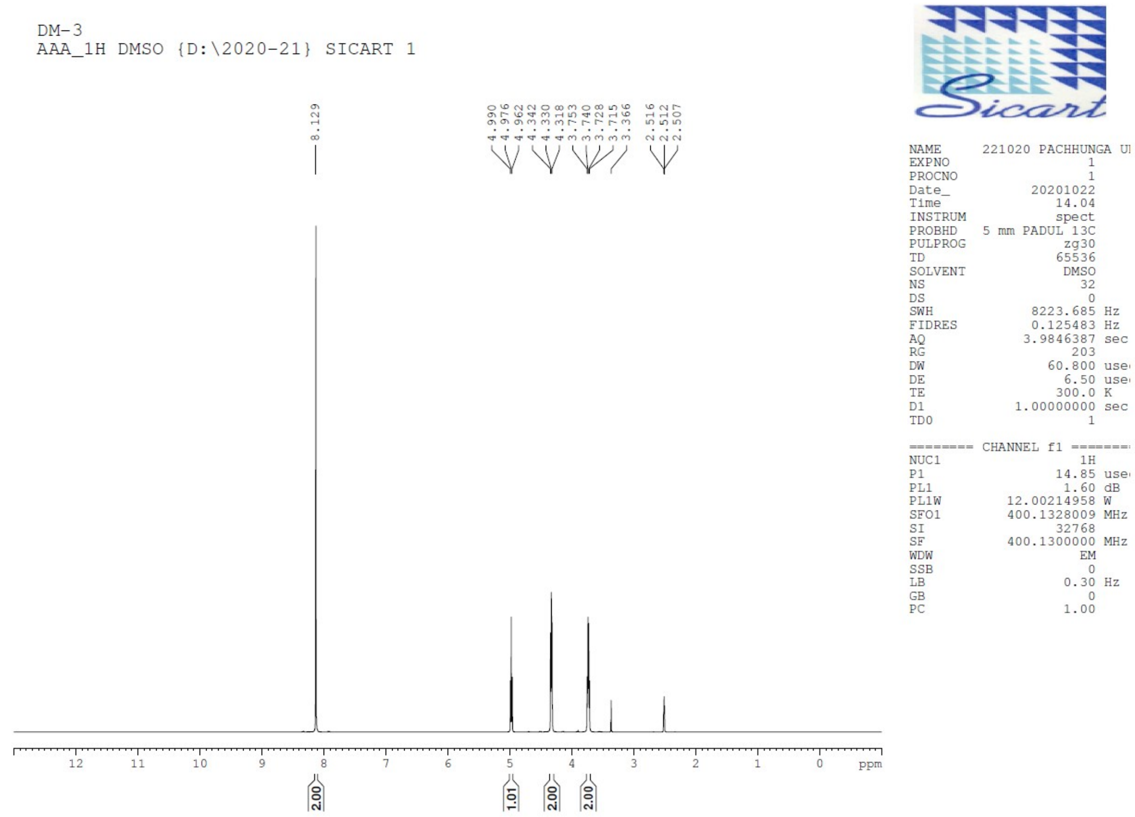

Figure S7: ^{13}C NMR spectra of BHET

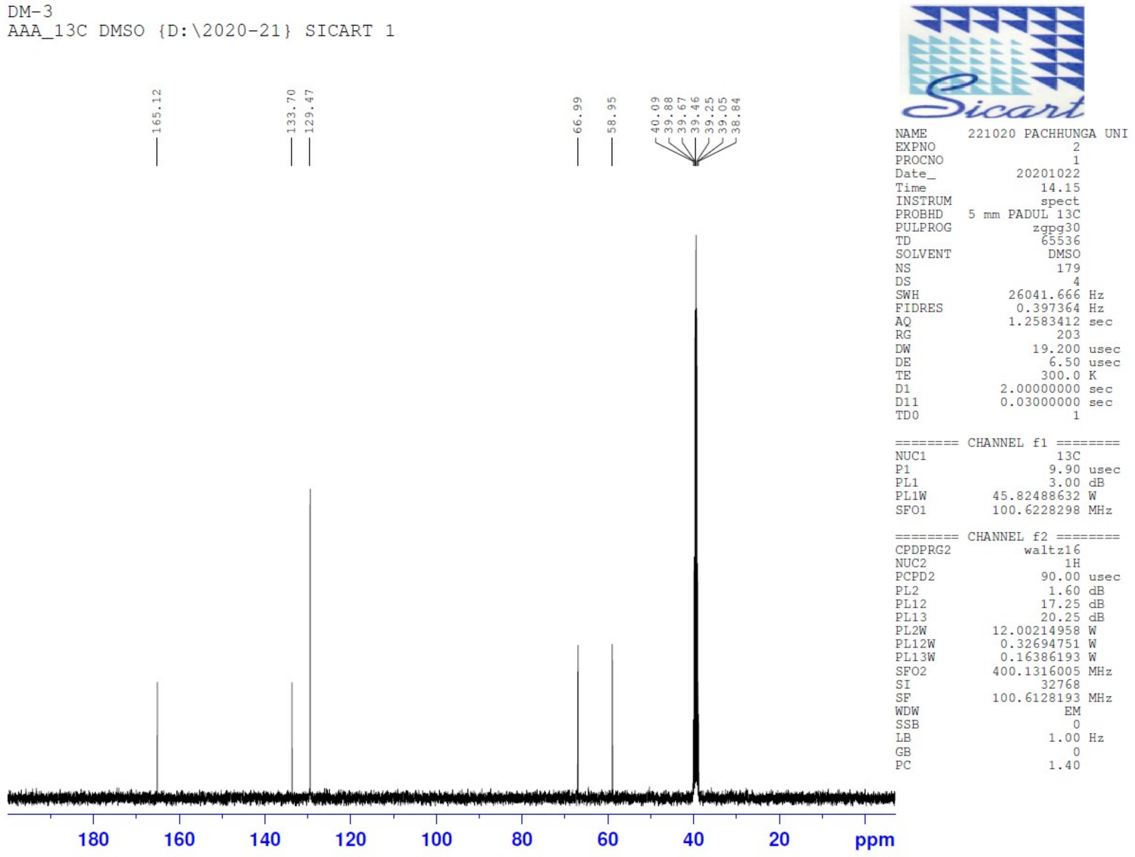

Figure S8: ^{1}H NMR data of water insoluble part

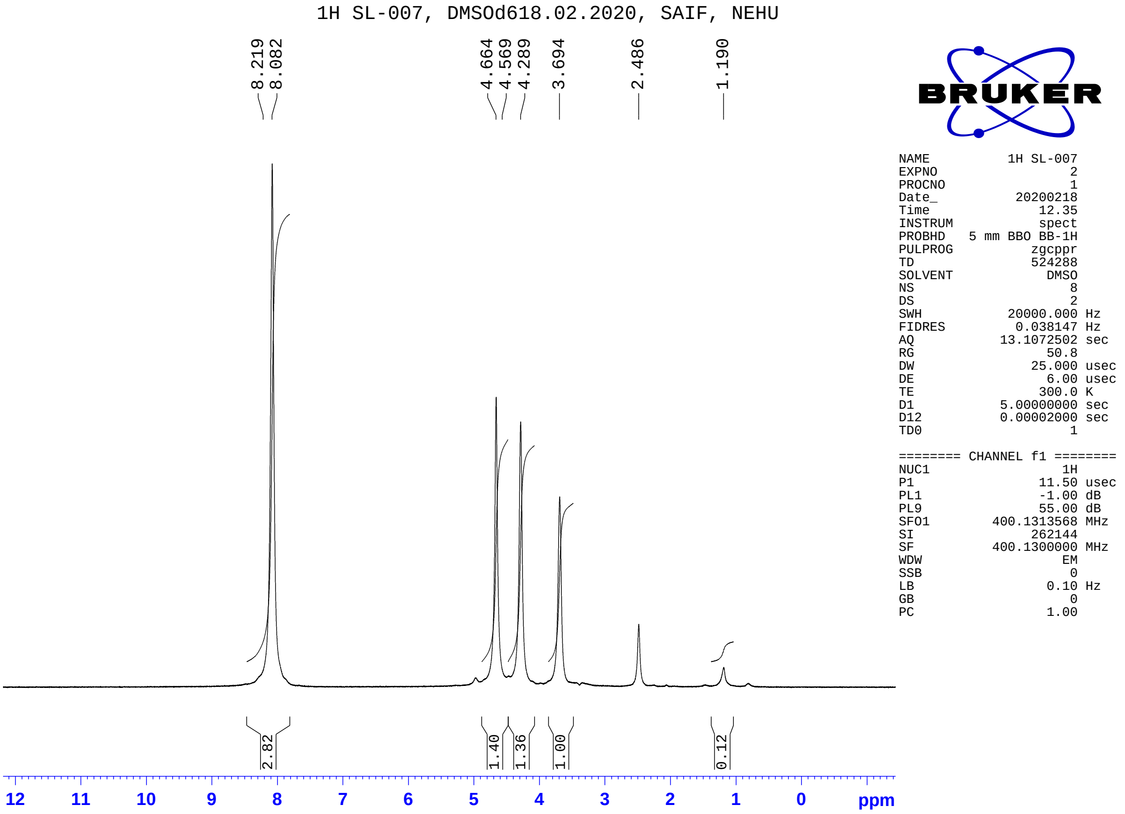

Figure S9: ^{13}C NMR data of water insoluble part

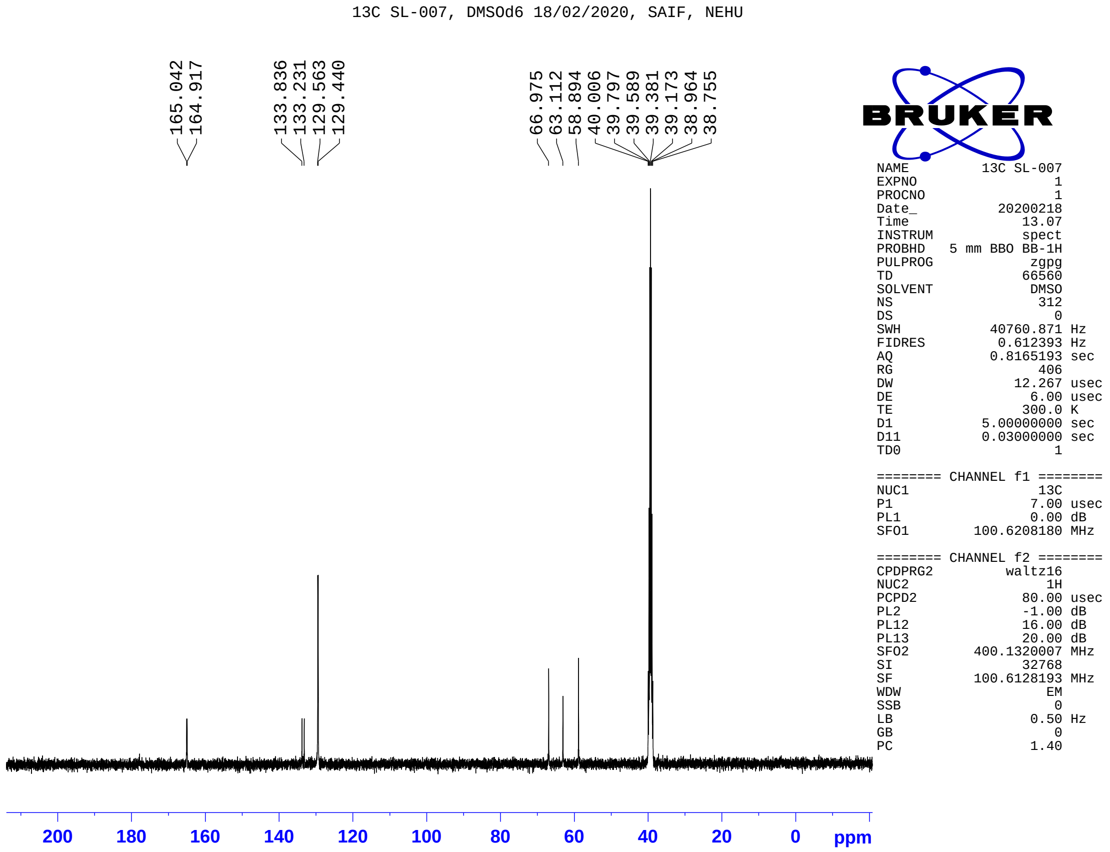

Figure S10: IR spectra of recrystallized BHET

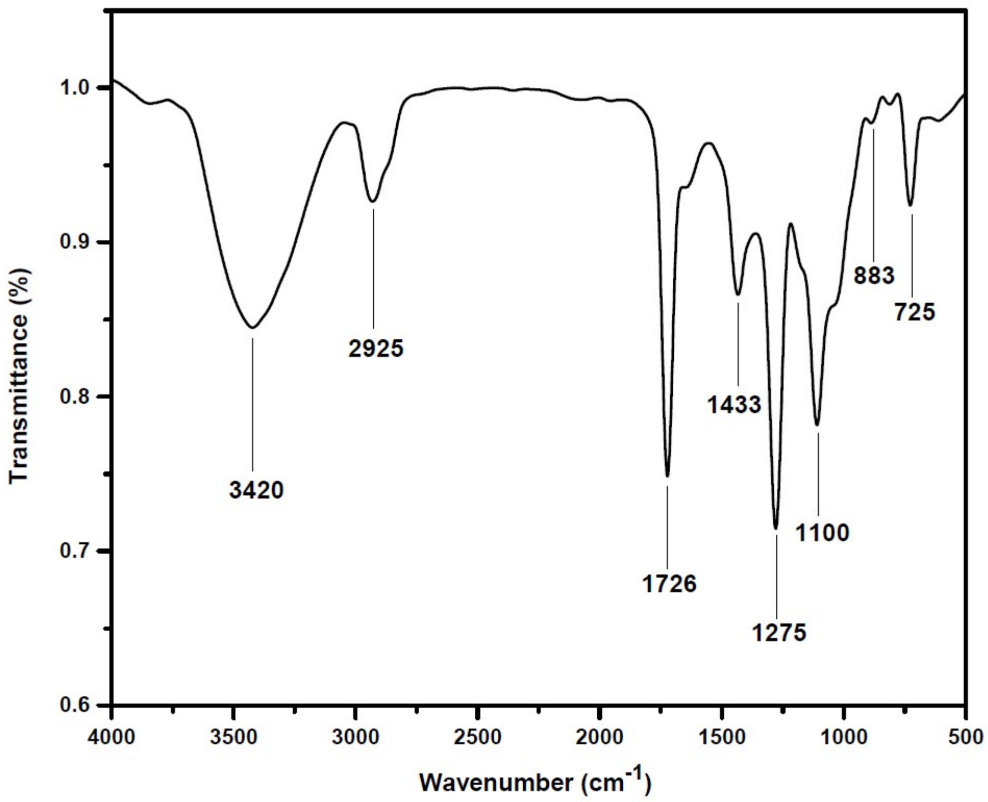

Figure S11: IR spectra of recovered OPA catalyst after the 5th cycle

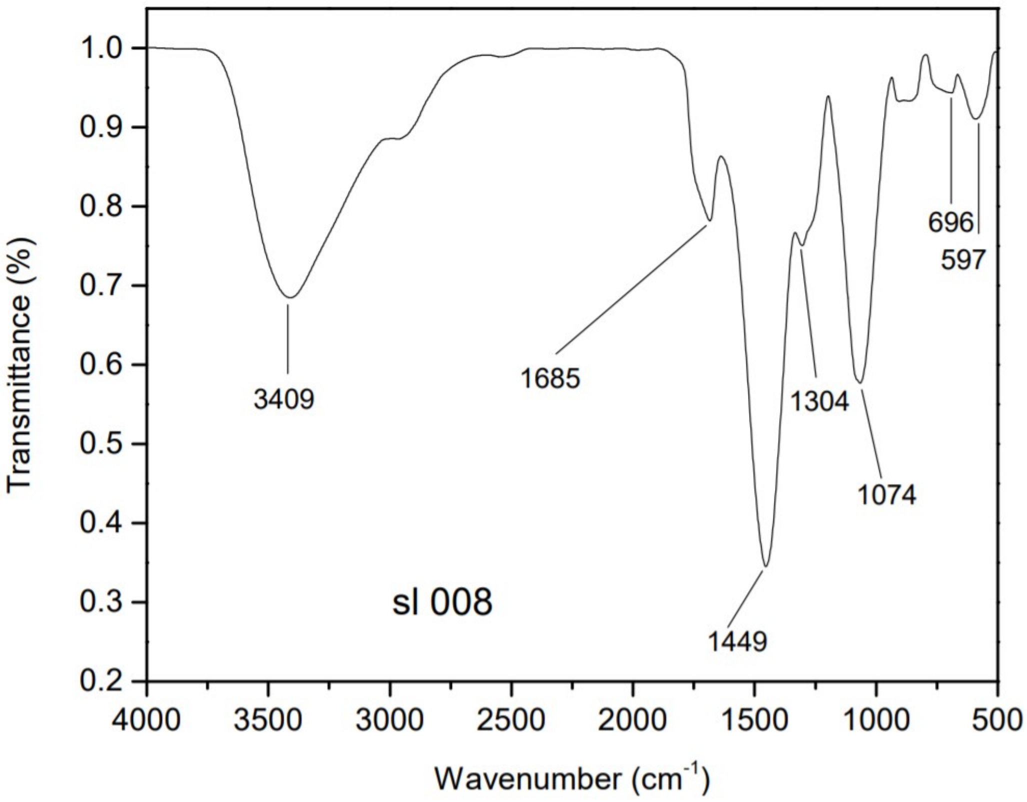

Figure S12: EDX data of recovered catalyst after the 5th cycle

EDAX TEAM

Full Area 1

kV: 20	Mag: 3500	Takeoff: 36.8	Live Time(s): 30	Amp Time(μs): 0.96	Resolution:(eV)135.5

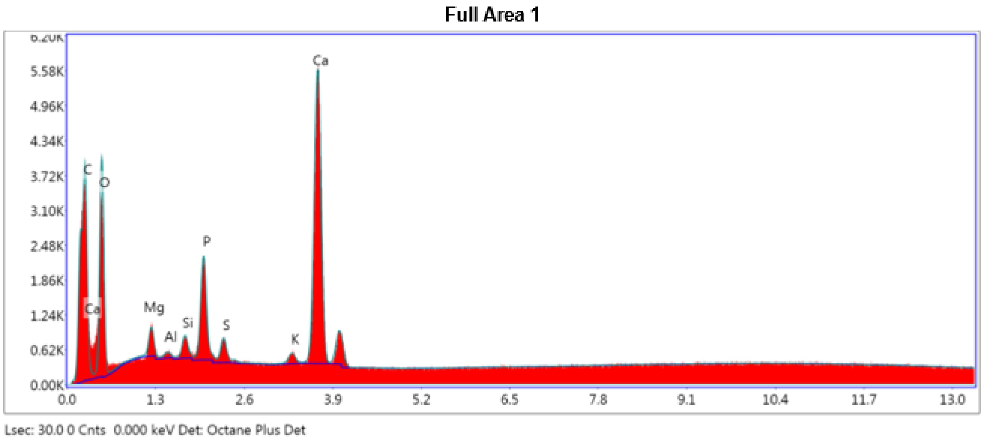

eZAF Smart Quant Results

Element	Weight %	Atomic %	Net Int.	Error %	Kratio	Z	R	A	F
C K	30.74	42.92	1,350.64	99.99	0.12	1.06	0.96	0.36	1
O K	42.94	45.00	1,801.18	9.92	0.07	1.02	0.98	0.15	1
MgK	1.29	0.89	305.21	8.89	0.01	0.94	1.02	0.5	1
AlK	0.17	0.11	51.16	32.88	0.00	0.91	1.02	0.65	1.01
SiK	0.68	0.41	240.92	9.01	0.00	0.93	1.03	0.77	1.01
P K	3.82	2.07	1,207.89	3.58	0.03	0.89	1.04	0.86	1.02
S K	0.84	0.44	278.42	8.99	0.01	0.91	1.04	0.89	1.02
K K	0.48	0.21	135.03	11.73	0.00	0.86	1.06	0.99	1.1
CaK	19.04	7.96	4,194.71	1.66	0.17	0.87	1.06	1	1

Figure S13: SEM images of recovered OPA after the 5th cycle

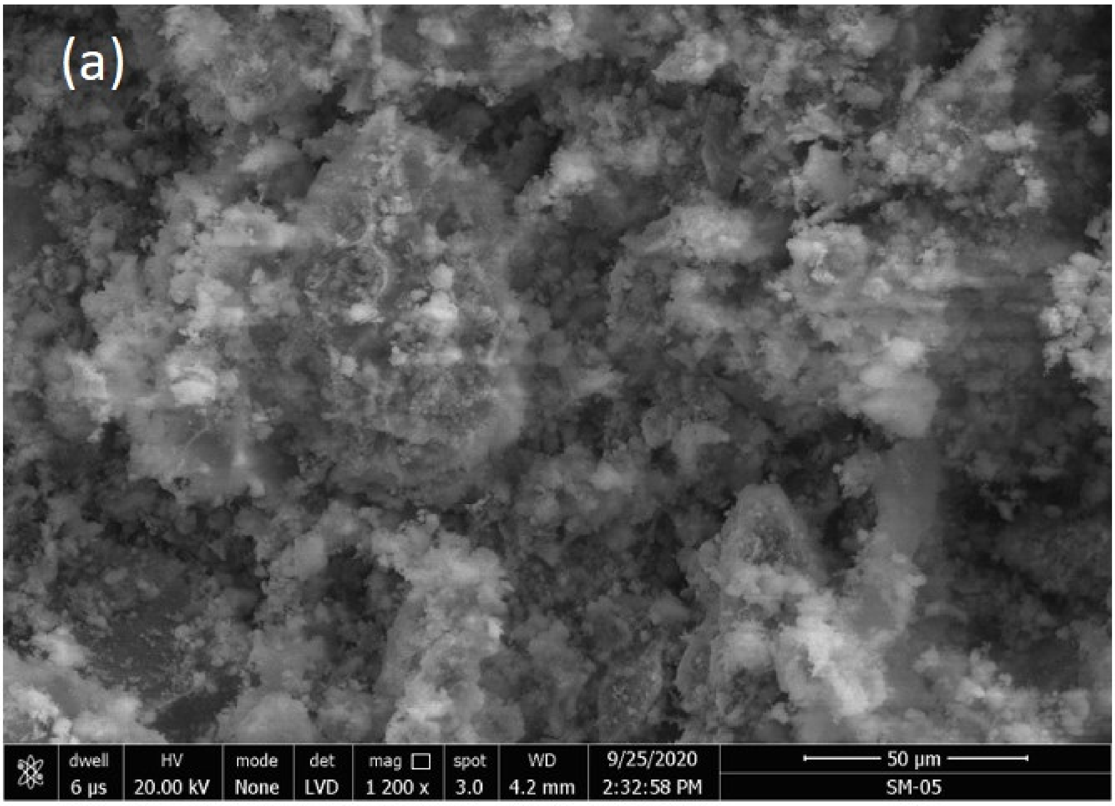

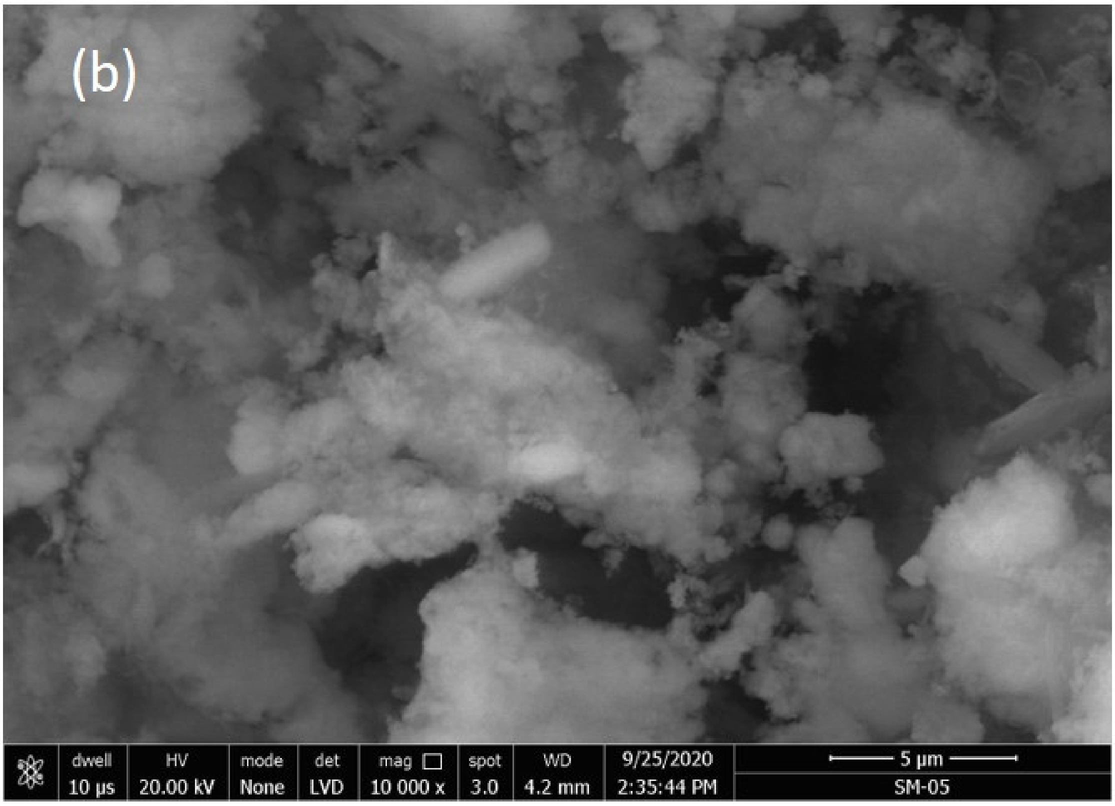

Figure S14: TEM image of recovered OPA after the 5th cycle

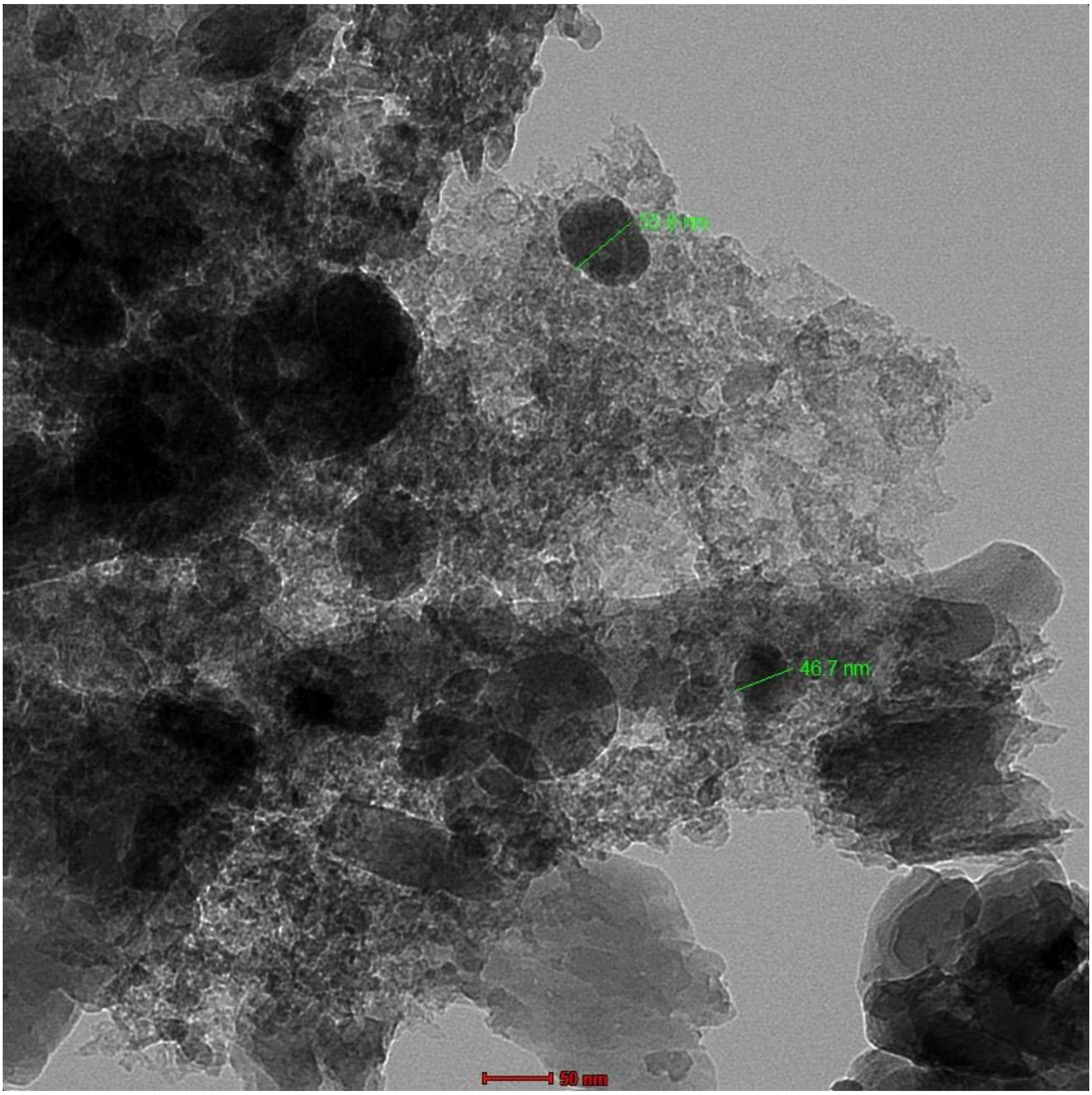

Supplement: Supplementary file 1 [file polymers-13-00037-s001.pdf]
